# Treatment intentions of general practitioners regarding hypertension in the oldest old: a vignette study

**DOI:** 10.1186/s12875-016-0523-y

**Published:** 2016-08-30

**Authors:** Evelyn Mermans, Jan Degryse, Bert Vaes

**Affiliations:** 1 Department of General Practice, Universiteit Leuven (KU Leuven), Leuven, Belgium; 2 Institute of Health and Society, Université catholique de Louvain (UCL), Brussels, Belgium

**Keywords:** Hypertension, Aged, 80 and over, General practitioners, Physical activity

## Abstract

**Background:**

The recent literature has shown that the risk of hypertension in the old strongly depends on their physical abilities. However, it is unknown whether general practitioners (GPs) adapt their treatment strategies to the patient’s independence. This study was conducted to investigate the treatment intentions of GPs for patients aged 80 and older with hypertension in relation to the patients’ level of dependency.

**Methods:**

A vignette study in Belgium. Flemish GPs (*n* = 305) were invited, directly or indirectly, by email to fill out a questionnaire, consisting of nine cases (three themes). In each theme, the level of dependency gradually increased. Per case, a score depending on the GP’s treatment intention was calculated (range 0–3). The total score represented the ‘Intention to Treat Hypertension in Older Persons’ scale (ITHOP-scale). The difference between the score for robust patients and strongly dependent patients was calculated (delta score).

**Results:**

The scores on the ITHOP scale showed a mean of 15.2 ± 6.0. A significant difference in treatment intention was found between robust patients and strongly dependent patients. The delta score showed a mean of 1.7 ± 1.8. Differences between the GPs were responsible for 75 % of the variance of the total score, and differences in the level of dependency did not influence the variance (G coefficient =0.82). The GP’s experience showed an inverse relationship to the total and the delta score.

**Conclusion:**

Large differences in treatment intentions for hypertension in the very old exist between GPs, but the patient’s level of dependency is not responsible for these differences.

**Electronic supplementary material:**

The online version of this article (doi:10.1186/s12875-016-0523-y) contains supplementary material, which is available to authorized users.

## Background

The Western world is facing a grey epidemic. In 2011, the life expectancy in Belgium was 78 years for men and 83 years for women [1]. By 2060 this will be 87 and 89 years, respectively. These demographic changes will have an important impact on the organisation of health care. The complexity of multimorbidity and polypharmacy in the old will be a permanent challenge for general practitioners (GPs) [2].

Cardiovascular morbidity and mortality remain important problems in the oldest old [3]. The management of the cardiovascular risk profile has been extensively explored [4]. The diagnosis and treatment of hypertension are central issues within this approach [5]. However, based on the current guidelines, information on patients aged 80 and over is limited [6]. Furthermore, contradictions exist regarding the risk of hypertension related to cardiovascular morbidity and mortality in the oldest old [7, 8]. No clear answer has been proposed from the different studies that have been performed, and major surveys with unambiguous conclusions are lacking.

The Hyvet study showed that antihypertensive treatment reduced stroke mortality as well as total mortality [9]. However, this study only included relatively healthy patients, and only a small subgroup was from Western Europe. In contrast, the meta-analysis by Musini et al. reported that antihypertensive treatment did not reduce total mortality in patients aged 80 and older [10]. Moreover, in the Leiden 85-plus study, patients with a lower blood pressure showed an increased risk of mortality compared to patients with hypertension [11].

The heterogeneity of the oldest old most likely plays an important role in the explanation of the differences in the risk of hypertension for cardiovascular and total mortality. A recent publication found that the association between blood pressure and mortality depended on the physical abilities, in particular the walking speed, of the study subjects. In slower walking adults, no association between an elevated systolic or diastolic blood pressure and total mortality was found [12]. To date, it is unknown whether GPs determine their treatment strategies for hypertension in the oldest old based on the patients’ level of dependency.

Therefore, this survey sought to explore the differences in the treatment intentions between GPs for hypertension in patients aged 80 and older and to investigate whether their treatment intentions depended on the patients’ level of dependency.

## Methods

### Recruitment of the GPs

In Belgium there is no general database of email addresses of GPs. Two strategies were used to invite GPs. First, email addresses of potential GPs were obtained from different GP societies and the National Order of Physicians. Second, when email addresses were protected for privacy reasons, the presidents of local GP associations were asked to forward our invitation to their members. In total a potential of some 5000 practicing Flemish (Flanders = northern Dutch-speaking part of Belgium) GPs or GPs in training (under the age of 70) were invited to participate in the survey. All contacts received the invitation twice by email.

For each participating GP, the following data were recorded: age, gender, function (GP, GP trainer, GP trainee or coordinating and consulting doctor in a nursing home), region (rural/urban), and years of practice (experience).

Following standard procedures at the University of Leuven (Belgium) the ethical review board of the Medical Faculty of the University of Leuven approved the study. When entering the electronic survey, all participating GPs were asked to give informed consent.

### Development of a scale

The electronic survey contained nine case vignettes. The case vignettes were split into three themes: the patient living at home without important comorbidity (case 1, 2, 3), the cardiovascular patient (case 4, 5, 6) and the patient with cognitive decline (case 7, 8, 9). Each vignette described a patient of at least 80 years old, with or without a previous history of arterial hypertension. All cases presented with an average systolic blood pressure >170 mmHg. In the three consecutive cases of one theme, the level of dependency progressively increased (from robust to moderately and strongly dependent) (See Additional file 1: Appendix 1). For each case, the same questions were asked (See Additional file 1: Appendix 2).

Each respondent received a score for each case. This score (range 0–3) was based on the treatment intention of the respondent. The lower the score, the less the respondent had the intention to change the antihypertensive treatment. A score of 3 was given when the GP strove for a strict antihypertensive treatment, and the target systolic blood pressure was lower than 140 mmHg. If the respondent chose to start a new antihypertensive medication with a target systolic blood pressure ≥140 mmHg, he received a score of 2. If only the dose of the present medication was increased, the respondent received a score of 1. Finally, if the antihypertensive treatment was not changed, the respondent received a score of 0. The total score (range 0–27) was calculated per respondent and represented the ‘Intention to Treat Hypertension in Older Persons’ scale (ITHOP scale). Subscores were for the different clusters of cases with the same level of dependency (robust (case 1, 4, 7), moderately dependent (case 2, 5, 8) and strongly dependent (case 3, 6, 9)).

First, the electronic survey was evaluated by 5 GPs in order to assess its face- and content validity. The definitive survey was performed from 15^th^ October 2013 until 20^th^ December 2013.

### Data analysis

The mean scores and subscores and their 95 % confidence intervals (CIs) are presented with error bars. The differences between the subscores were investigated with the paired *t* test. For each respondent, the difference in the treatment intention according to the level of dependency was defined as the difference (delta score) between the subscore for the robust cases and the subscore for the strongly dependent cases. The internal consistency of the scale was assessed by means of Cronbach’s alpha coefficient.

A generalisability analysis was performed to analyse the contribution of the several potential sources of error in the measurements [13–15]. Generalisability theory offers a framework to estimate the magnitude of the multiple sources of error and to assess the reliability of measurements tailored to specific clinical applications. The theory offers a framework in which these different conditions can be related to each other to subsequently assess their impact or contribution to the reliability of the tests [16]. A p x (c:d) design was used (*p* = persons c = cases and d = level of dependency).

Linear regression analysis was performed with the total score as the dependent variable, and gender, function, region and experience (or age) as independent variables. Next, linear regression analysis with the delta score as the dependent variable and the total score, gender, function, region and experience (or age) as the independent variables.

A random sample, adjusted for age and gender according to the general Flemish GP population was created and a sensitivity analysis was performed (Additional file 1: Appendix 3).

The data analysis was performed with SPSS 22.0 for Windows (SPSS Inc., Chicago, IL, USA) and GENOVA software (University of Iowa, Iowa City, IA, USA).

## Results

### Respondent characteristics

A total of 409 GPs opened the survey, of which 293 finished the survey completely. For those who finished at least six cases (*n* = 12), the missing scores were replaced by the respondent’s average score of the first two cases with the same level of dependency. The final analyses were performed for a total of 305 respondents. The background variables of the GP’s are reported in Table 1. In general GPs were more often female and younger than the average Flemish GP (Additional file 1: Appendix 3) [17].

**Table 1:** Responder characteristics (*n* = 305)

Male, n (%)	157 (52.5)
Age (years), n (%)	
25–35	105 (34.7)
36–45	32 (10.6)
46–55	78 (25.7)
56–65	74 (24.4)
>65	14 (4.6)
Experience (years), n (%)	
<5	84 (27.7)
5–10	36 (11.9)
11–20	25 (8.3)
21–30	73 (24.1)
>30	85 (28.1)
Job position, n (%)	
Trainee	33 (10.8)
Trainer	20 (6.6)
CCD	15 (4.9)
Other	241 (79.0)
Location practice, n (%)	
City	135 (44.3)
Rural	137 (44.9)
Other	34 (11.1)

*CCD* coordinating and consulting doctor in a nursing home

### Descriptive statistics

**Fig. 1:**
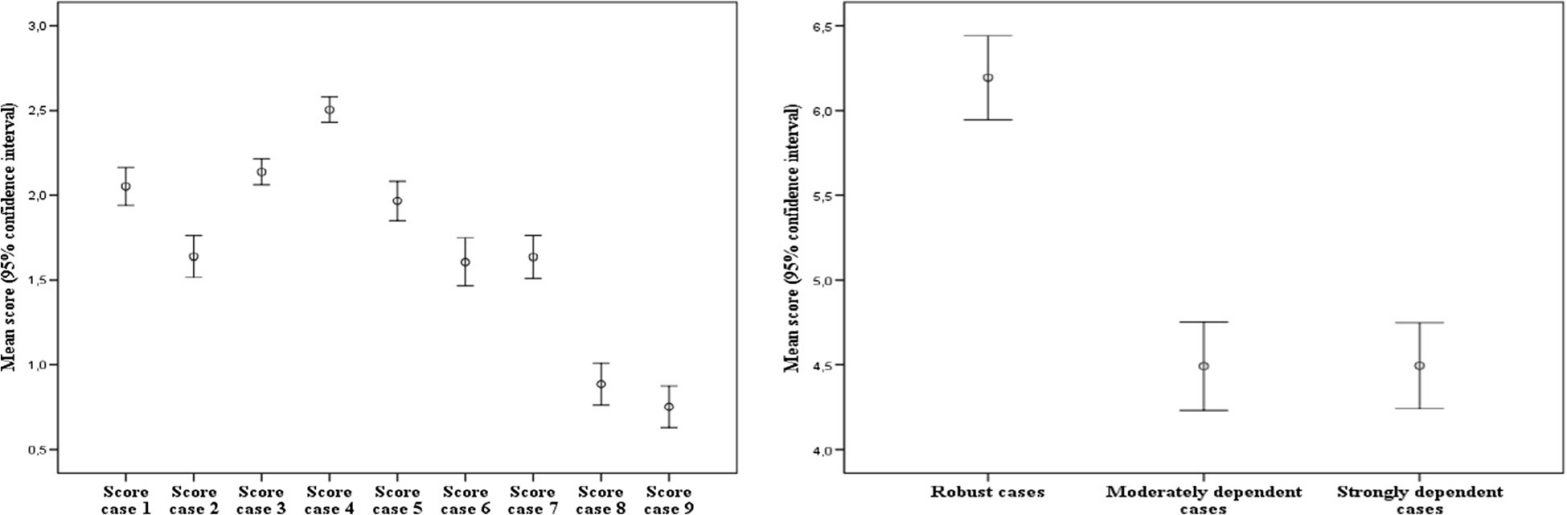
Error bars of the mean scores and 95 % confidence intervals of separate cases and clusters of cases according to the level of dependency. **a** (*left*) and **b** (*right*). Legend: **b**: Robust: sum of cases 1, 4 and 7; Moderately dependent cases: sum of cases 2, 5 and 8; Strongly dependent cases: sum of cases 3, 6 and 9

The scores on the ITHOP scale were normally distributed and ranged from 0 to 27 with a mean (± standard deviation (SD)) of 15.2 ± 6.0. Cronbach’s alpha of the scale was 0.83. Figure 1a shows the mean case scores. A trend break was observed in case 2. Figure 1b shows the mean cluster scores of cases with the same level of dependency. The mean subscores were 6.2 ± 2.2, 4.5 ± 2.3 and 4.5 ± 2.3 for the cluster with robust patients and the clusters with moderately and strongly dependent cases, respectively. A significant difference was found between the mean subscore for robust cases and the subscores for moderately and strongly dependent cases (*P* < 0.001). No difference was found between the subscores for moderately and strongly dependent cases (*P* = 0.98). The delta score ranged from −3 to 7 with a mean score of 1.7 ± 1.8.

### Generalisability analysis

The generalisability analysis produced a reproducibility index of 0.82 for the total score (Table 2). This was used to calculate a SEM $\left(\mathrm{Standard}\ \mathrm{Error}\ \mathrm{of}\ \mathrm{Measurement} = \mathrm{S}\mathrm{D} \times \sqrt{1-0,82}\right)$ of 2.47 points with a 95 % CI (=1.96 × SEM) of 4.85 points. The differences between respondents (p) accounted for 75 % of the variance in the total score, whereas differences in the level of dependency (d) did not contribute. The c:d component reflects differences of cases within the same level of dependency and accounted for 7.6 % of the variance in the total score. The pxd effect denotes the ways in which GPs manage hypertension differently in cases with other levels of dependency and did not contribute to the variance in the total score. Finally, the pc:d effect denotes the ways in which GPs manage hypertension differently in other cases with the same level of dependency and explained 16 % of the variance in the total score.

**Table 2:** Generalisability analysis to analyse the contribution of the several potential sources of error in the score on the ITHOP scale (p x (c:d))

Effect	Variance component	Standard error	Percentage of the total variance
p	0.36219	0.03593	75.7
d	0.00000	0.03115	0
c:d	0.03627	0.01826	7.6
pxd	0.00000	0.00451	0
pc:d	0.07994	0.00265	16.7

A reproducibility index (G coefficient) of 0.82 was found for the total score

*ITHOP*, intention to treat hypertension in older persons, *p* persons, *c* cases, *d* degree of dependency

### Multivariate analysis

The multivariate analysis (Table 3) showed that the experience or the age of the respondents and the location of the practice were independent predictors of the total score. A correlation coefficient of 0.95 was found between the age and the experience of the respondents. Therefore, these variables were not included simultaneously in the multivariate analysis. Experience or age showed an inverse relationship to the total score (ß = −0.59 (95 % CI −1.0, −0.14), *P* = 0.010). The delta score also showed an inverse relationship to the experience or the age of the respondent (ß = −0.14 (95 % CI −0.27, −0.011), *P* = 0.034). No relationship was observed between the delta score and the total score (β = −0.006 (95 % CI −0.041, 0.028), *P* = 0.72). There was no multivariate analysis performed for the delta score.

**Table 3:** Linear regression analysis to identify determinants of the score on the ITHOP scale

	Bivariate		Multivariate	
	β (95 % CI)	*P* value^μ^	β (95 % CI)	*P* value
Male	−0.16 (−1.5, 1.2)	0.82	-	-
Age (categories)^a^	−0.63 (−1.1,−0.12)	0.015	-	-
Experience (categories)^a^	−0.47 (−0.89,−0.045)	0.030	−0.59 (−1.0,−0.14)	0.010
Job position				
Trainee	−0.066 (−2.2, 2.1)	0.95	-	-
Trainer	−1.8 (−4.5, 0.92)	0.19	-	-
CCD	0.23 (−2.9, 3.6)	0.88	-	-
Location practice				
City	−1.5 (−2.8,−0.14)	0.030	0.78 (−1.6, 3.1)	0.52
Rural	1.7 (0.36, 3.0)	0.013	2.6 (0.24, 5.0)	0.031

^a^Correlation coefficient is 0.95 (*P* < 0.001): the variables were not entered together in the multivariate analysis; ^μ^Determinants with a *P* value <0.10 were entered in the multivariable model

*ITHOP* intention to treat hypertension in older persons, *CCD* coordinating and consulting doctor in a nursing home

### Sensitivity analysis

Because of the overrepresentation of younger and female GPs in this study population an age and gender adjusted subsample was selected at random (*n* = 158) to perform a sensitivity analysis. The analyses on the subsample showed the same results compared to the analyses on the entire population (See Additional file 1: Appendix 3).

## Discussion

This study explored the differences in the treatment intentions of GP’s regarding old patients with hypertension. The “Intention to Treat Hypertension in Older Persons” (ITHOP) scale was shown to be reproducible with a G coefficient of 0.82. The variations in the treatment intentions were mainly attributed to the differences between the GPs and not to differences between the cases (themes) or to the differences in the level of dependency. An inverse relationship was found between the experience or age of the GP and the total score, but the delta score also showed an inverse relationship to the experience of the GP. Thus, the older the physician, the lower the overall treatment intention, and the smaller the difference between the treatment intentions for robust patients and strongly dependent patients.

The current findings are consistent with what has been previously described regarding the assessment of medical competence as “case-specificity”, which means that scores on a particular case poorly predict scores on another case (or item) in a test. The intention to adapt a treatment or not appears to be highly context dependent [18]. The mean subscore for robust patients differed significantly from the mean subscores for moderately and strongly dependent patients in our study. Moreover, differences in the patients’ level of dependency were not responsible for the variation in the overall treatment intention. This is also consistent with previous epidemiological findings that showed a lower mortality rate in hypertensive patients with a higher degree of frailty. Hypertensive patients with a slow walking speed, which was used as an indicator of frailty, showed a lower mortality rate than the robust hypertensive patients [12]. Furthermore, Sabayan et al. observed less physical and cognitive decline in hypertensive patients, especially in older patients with physical limitations [19]. Moreover, Ni Chróinín et al recently showed, using a case vignette study, that geriatricians more often deprescribe medications, like antihypertensives, in the setting of advancing dependency and cognitive impairment [20]. However, recently an analysis of the Hyvet trial did not show an interaction between frailty as measured by a frailty index (based on 60 deficits) and the effect of treatment for hypertension in adults aged 80 years and above [21]. And Moonen et al. found that discontinuation of antihypertensive treatment in older persons with mild cognitive deficits did not improve cognitive, psychological or general daily functioning after 16 weeks of follow-up [22].

Differences in clinical experience between the GPs were observed as a possible explanation for the differences in treatment intentions. The current analyses showed that younger or less experienced physicians had an overall higher treatment intention than older or more experienced doctors. As physicians age, they seem to use less stringent treatment targets or become less aggressive in reaching the defined goals. Possibly, older doctors are more receptive to a negative perception of the ageing process. On the other hand, the treatment strategies of more experienced physicians seem to be less influenced by the level of dependency of their patients. Patients with a higher level of dependency were treated even more intensively by older GPs than by younger GPs. Possibly, younger GP’s are more familiar with concepts such as ‘patient-centered’ and ‘goal-oriented’ care, as a result of their more recent graduation [23]. Older doctors may make less of a distinction between older patients because of their own experiences with the ageing process.

This study is the first survey that investigated the treatment intentions of GPs for patients aged 80 and over with hypertension. The ITHOP scale that was embedded in the survey appeared to have a high internal consistency and the G-analysis confirmed the reliability of the scores produced by the scale. The interindividual differences probably reflected the absence of guidelines on the treatment of hypertension in the oldest old and highlighted the importance of future research on these issues. Moreover, this study was the first to examine the impact of the level of the patient’s dependency on the GP’s treatment intention.

The present study showed that differences in treatment intentions for hypertension in older patients are primarily associated with differences between GPs. However, to date, it is unclear whether these differences are related to a difference in knowledge or a difference in attitude. This indicates the need for additional qualitative research that may bring more clarity regarding these issues. Moreover, there are no guidelines for the management of hypertension in those aged 80 and over.

A few limitations should be considered. First, participating GPs were more often female and younger than the average Flemish GP, making these results less representative for Flemish GPs [17]. However, a sensitivity analysis was performed on an age and gender adjusted subsample and showed the same results as the analyses in the total population. Second, although the presented cases had high face validity, it was impossible to fully display the complete nuance of the patient’s level of dependency. This could have led to a different interpretation of the respondents, which possibly explains the trend break in Fig. 1a.

## Conclusion

A significant difference in the treatment intention of GPs for people aged 80 and over with hypertension was found between robust patients and strongly dependent patients. The treatment intention was significantly higher in robust cases versus moderately or strongly dependent cases. On the other hand, large differences in overall treatment intentions were observed and could be mainly explained by differences between GPs and not by differences between cases or by differences in the patient’s level of dependency. This study underscores the need to develop adapted guidelines for the management of hypertension in the oldest old.

## Additional files

Additional file 1: Appendices. **Appendix 1**: Example of the case vignettes. **Appendix 2**: Questions asked in each case. **Appendix 3**: Sensitivity analysis. (DOCX 125 kb)

## Abbreviations

CI: Confidence interval
GP: General practitioner
ITHOP scale: Intention to treat hypertension in older persons scale
SD: Standard deviation
SEM: Standard error of measurement
