# Supplementary material for: Treatment intentions of general practitioners regarding hypertension in the oldest old: a vignette study
Source: BMC Fam Pract. 2016 Aug 30;17(1):122. doi: 10.1186/s12875-016-0523-y (PMC5006442; doi:10.1186/s12875-016-0523-y)
Supplement: Additional file 1: — Appendices. Appendix 1: Example of the case vignettes. Appendix 2: Questions asked in each case. Appendix 3: Sensitivity analysis. (DOCX 125 kb) [file 12875_2016_523_MOESM1_ESM.docx]

Appendix 1. Example of the case vignettes

1. Robust: Case 1

Mr Lemmens is 85 years old. You have seen him only a few times on consultation. He is an active old man and goes everywhere by foot. Only for longer distances, he walks with a walking stick. He is able to manage his household by himself. He’s a widower and lives alone now for 20 years.

His medical history:

- 1990: gout
- 1995: hypertension

His medication: Bisoprolol 5 mg, 1x/day - Acetylsalicylic acid 80 mg, 1x/day - Lormetazepam 1mg, 1x/day

He comes to visit you to ask for new prescriptions.

Your physical examination:

Pulse: 75/min; BP: 170/100mmHg

Heart: regular rhythm

Lungs: normal breathing sounds

Five minutes later, you measure the BP again: 170/95mmHg

Two weeks later, you ask him to come back to control his BP. Again, his BP is high, 175/90mmHg.

1. Moderately dependent: Case 5

Mr Claessens is 82 years old. He lives in a service flat across the street. His daughter does the shopping. For more than a year a nurse passes every week to prepare his medication. Since last week the nurse passes every day because of a venous ulcer on his left leg.

His medical history:

- 1998 Diabetes mellitus type 2 (last HbA1c = 6.8%)
- 2000 Coronary artery bypass graft
- 2005 mild left ventricular dysfunction
- Last estimated glomerular filtration rate (eGFR) was 65 ml/min/1,73m²

His medication: Bisoprolol 5 mg, 1x/day - Bumetanide 1 mg, 1x/day - Pravastatin 20 mg, 1x/day - Gliquidone 30 mg, 1x/day – Insulin - Acetylsalicylic acid 80 mg, 1x/d

Because of his cardiac problems, he regularly measures his blood pressure at home. Last few weeks the blood pressure was high: 190/100mmHg - 180/85mmg - 190/90mmHg - 185/95mmHg.

1. Strongly dependent: case 9

Mrs Luijten is 85 years old. She lives at home with her husband, who takes care of her. A nurse passes twice per day to wash and (un)dress her. Her daughter passes a few times every day. The continuous care is difficult for her husband, but he does not want her to be institutionalized in a nursing home. There is little verbal communication possible. Sometimes she is sitting in a chair, but mostly she is in bed.

Her medical history:

- 2003: Alzheimer's disease
- 2005: atrial fibrillation
- 2011: fracture of the right femur after a fall from the bed, treated with a total hip prosthesis.
- 2012: Pneumonia because of swallowing disorders in the context of dementia.

Her medication: Paracetamol 600 mg suppositories, 4x/day - Sotalol 2x80mg/day - Glycerin suppositories, if necessary - Clopidogrel 75mg, 1x/day - Ranitidine 150mg/10 ml syrup, 2x/day -Calcium 1g + colecalciferol 880IE, 1x/day

The last blood pressure measurements were increased: 180/90mmHg - 185/100mmHg - 170/80mmHg - 175/85mmHg

Appendix 2. Questions asked in each case.

1. Do you want to adjust the antihypertensive treatment for this patient? Yes/No, because?
2. How would you adjust the antihypertensive treatment for this patient?
   1. By starting a new antihypertensive drug
   2. By raising the dose of a present antihypertensive drug
   3. By changing one antihypertensive drug by another
3. Which antihypertensive drug would you then chose for this patient? Please, give a top 3.
   1. Thiazide
   2. Loop diuretic
   3. Beta-blocker
   4. Calcium antagonist
   5. Angiotensin converting enzyme inhibitor
   6. Angiotensin receptor blocker
   7. Potassium-sparing diuretic
   8. Other
4. What is your target systolic blood pressure for this patient?
   1. <120 mmHg
   2. 120-140 mmHg
   3. 140-160 mmHg
   4. >160 mmHg

Appendix 3. Sensitivity analysis

When the gender and age distribution of our sample was compared with the Flemish GP population we observed a small overrepresentation of female GPs in the total group. Women represent 40% of the total GP population in Flanders and Brussels [18]. Furthermore, mainly younger GPs (25 – 35 years) were overrepresented in our sample (Table 1 sensitivity analysis). Therefore, an age and gender adjusted subsample of the total population was selected at random (n = 158) to perform a sensitivity analysis.

| Table 1 sensitivity analysis. Age and gender distribution of general practitioners in Flanders, the total study population and an adjusted random sample of the study population | | | | | | |
| --- | --- | --- | --- | --- | --- | --- |
|  | General practitioners in Flanders (Belgium) | | Total study population (n = 305^*^) | | Adjusted random sample for sensitivity analysis (n = 158) | |
|  | Women  (40%) | Men  (60%) | Women  (n = 142, 47%) | Men  (n = 157, 51%) | Women  (n = 63, 40%) | Men  (n = 95, 60%) |
| 25 – 35 years | 20.4% | 4.7% | 77 (54.6) | 28 (17.8) | 13 (20.6) | 5 (5.3) |
| 36 – 45 years | 33.9% | 9.7% | 23 (16.3) | 9 (5.7) | 21 (33.3) | 9 (9.5) |
| 46 – 55 years | 30.0% | 22.5% | 28 (19.9) | 49 (31.2) | 19 (30.2) | 21 (22.1) |
| 56+ years | 15.7% | 63.1% | 13 (9.2) | 71 (45.2) | 10 (15.9) | 60 (63.1) |
| ^*^, Age or gender was not registered by 7 respondents (2%). | | | | | | |

Results of the sensitivity analysis:

In the total study population the scores on the ITHOP scale were normally distributed and ranged from 0 to 27 with a mean score of 15.2 ± 6.0. In the adjusted random sample (n = 158) the scores on the ITHOP scale also showed a normal distribution and ranged from 0 to 27 with a mean score of 14.8 ± 6.1. Cronbach’s alpha of the scale in the adjusted random sample was 0.84.

| Figure 1 sensitivity analysis. Error bars of the mean scores and 95% confidence intervals of separate cases and clusters of cases according to the level of dependency (n = 158) | |
| --- | --- |
| Figure A sensitivity analysis | Figure B sensitivity analysis |
| 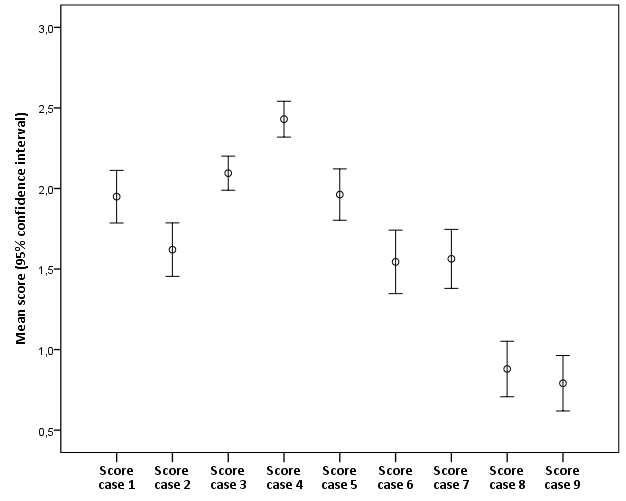 | 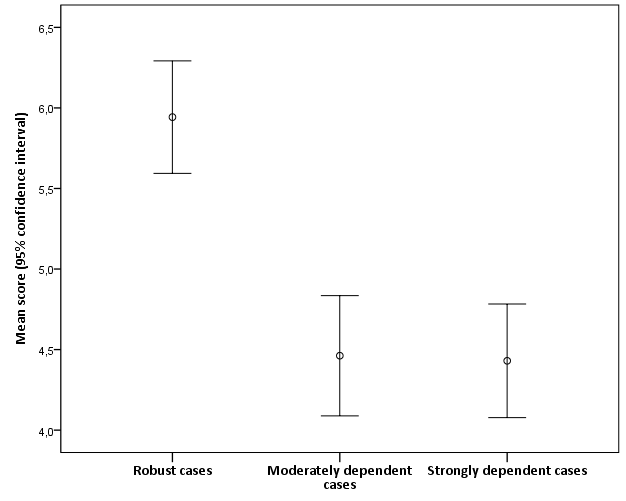 |
| Figure B: Robust: sum of cases 1, 4 and 7; Moderately dependent cases: sum of cases 2, 5 and 8; Strongly dependent cases: sum of cases 3, 6 and 9. | |

When Figure 1 was reproduced with the adjusted random sample, the same pattern was seen (Figure 1 sensitivity analysis). In the total study population the mean subscores were 6.2 ± 2.2, 4.5 ± 2.3 and 4.5 ± 2.3 for the cluster with robust patients and the clusters with moderately and strongly dependent cases, respectively. In the adjusted random sample the mean subscores were 5.9 ± 2.2, 4.5 ± 2.4 and 4.4 ± 2.2, respectively.

In the total study population the delta score ranged from -3 to 7 with a mean score of 1.7 ± 1.8. In the adjusted random sample the delta score ranged from -2 to 7 with a mean score of 1.5 ± 1.8.

In the total population the generalisability analysis produced a reproducibility index of 0.82 for the total score. In the adjusted subsample a reproducibility index of 0.86 was found for the total score. Using this reliability index a SEM ($Standard Error of Measurement= SD*\sqrt{1-0,86})$ of 1.88 points and a 95% CI (= 1.96 x SEM) of 3.69 points was calculated.

Table 2 (sensitivity analysis) shows the results of the generalizability analysis that investigates the contribution of different variables to the total score and showed the same results as the generalizability analysis in the total population.

| Table 2 sensitivity analysis. Generalisability analysis to analyse the contribution of the several potential sources of error in the score on the ITHOP scale (p x (c:d)) | | | |
| --- | --- | --- | --- |
| Effect | Variance component | Standard error | Percentage of the total variance |
| p | 0.38062 | 0.05185 | 77.1 |
| d | 0.00000 | 0.02598 | 0 |
| c:d | 0.03369 | 0.01710 | 6.82 |
| pxd | 0.00000 | 0.00595 | 0 |
| pc:d | 0.07994 | 0.00368 | 16.17 |
| ITHOP: intention to treat hypertension in older persons; p = persons, c = cases, d = degree of dependency.  A reproducibility index (G coefficient) of 0.86 was found for the total score. | | | |

The multivariate analysis in the adjusted random sample showed the same trend as the analysis in the total population. The total score on the ITHOP scale showed an inverse relationship to the experience of the GP (β -0.90 (95% CI -1.6, -0.21), P = 0.011) and was correlated with the location of the practice (rural location, β = 2.0 (95% CI 0.13 – 3.9), P = 0.036). The delta score also showed an inverse relationship to the experience of the respondent (β = -0.26 (95% CI -0.47, -0.047), P = 0.017).
